# The influence of hypoxia and prolonged exercise on attentional performance at high and extreme altitudes: A pilot study

**DOI:** 10.1371/journal.pone.0205285

**Published:** 2018-10-03

**Authors:** Mirjam Limmer, Petra Platen

**Affiliations:** 1 Department of Sports Medicine and Sports Nutrition, Ruhr-University Bochum, Bochum, Germany; 2 Institute of Outdoor Sports and Environmental Science, German Sports University Cologne, Cologne, Germany; German Sport University, GERMANY

## Abstract

**Introduction:**

Exposure to hypoxic conditions is reported to impair cognitive performance. Further, moderate physical exercise improves cognitive function, but little is known about the influence of exercise on cognitive function in hypoxia. Therefore, the current study aimed to examine the influence of hypoxia (HYP) and prolonged exercise (EX) on attentional performance.

**Methods:**

A total of 80 participants (female: n = 29; male: n = 51) were assigned to four groups: HYP + EX (n = 15), HYP (n = 25), EX (n = 21) and normoxia (NOR) (n = 21). The Frankfurt Attention Inventory—2 (FAIR-2) was performed at four testing points (day 1, 14, 16 and 18) to assess attentional performance. All groups completed a pretest (D1) and a follow-up test (D18). In HYP + EX conditions, the cognitive task was performed in a hypoxic state after prolonged exercise (D14: 3950 m, D16: 5739 m) during a mountain climb on Mt. Kilimanjaro. Participants in HYP were tested under intermittent hypoxia at rest in a hypoxic chamber (D14: 3500 m, D16: 5800 m), and those in EX were tested under normoxia after prolonged exercise during a 7-day backcountry ski hiking tour. NOR was a control group, and participants completed all tests under normoxia and at rest.

**Results:**

Hypoxia impaired the attentional functions *performance value* (PV) and *continuity value (*CV) for the HYP + EX (p = 0.000) and HYP (L: p = 0.025; K: p = 0.043) groups at 5739 m and 5800 m, respectively, but not the function *quality value* (QV). In contrast, the EX group did not exhibit changes in attentional function.

**Conclusion:**

The current results suggest that attentional performance is impaired during extreme normobaric and hypobaric hypoxic exposure. We further conclude that greater cognitive impairment under hypobaric hypoxia during a mountain climb compared with normobaric hypoxia at rest is not caused by prolonged exercise, but may be influenced by other factors (e.g. low temperatures, dehydration, or sleep deprivation) that remain to be verified.

## Introduction

There is currently widespread interest in high-altitude activities internationally, including mountaineering, hiking and skiing [1]. During altitude exposure, the oxygen partial pressure decreases exponentially with increasing altitude and the resulting hypoxic conditions lead to cognitive and physiological alterations caused by decreased O_2_ intake [2,3]. In 1925, early mountaineers reported impairment of mental skills and a reduced ability to concentrate during a high altitude mountaineering expedition [4]. Recent studies investigating the effects of hypoxic conditions on cognitive functions have reported impairments in a range of cognitive abilities, such as memory [5,6], learning [7], reaction time [8,9] and decision making [6,7]. Cognitive impairment, particularly attentional dysfunction, has negative effects on high altitude mountaineering expeditions, including poor decision making and situation assessment, errors in perception of environmental events or cues, and distraction [2,10]. For instance, attention impairment and associated distraction from rope and safety management, weather observations, or changes in attention to climbing partners’ physical health could result in inappropriate decisions. Because of the risks associated with mountain conditions, impaired decision making can lead to falls, injuries, and death [10]. However, there is a small but increasing body of evidence regarding the effects of high altitude on attention [3]. One previous study reported an impairment of attention capacity at simulated altitudes between 3000 and 5000 m [11]. Additionally, impaired attention is described for acute exposure to simulated altitude (4500 m) at a maximum of 24 h [1,12] and for environmental hypobaric hypoxia up to 5300 m [13]. In contrast, one study reported attentional changes in mountaineers during a high altitude expedition involving a stay of 21 days at 6542 m, but not for a second group of mountaineers that stayed 18 days between 2000 and 6440 m [14].

Cognitive performance may also be impaired by other factors occurring during mountaineering and high altitude expeditions, such as strenuous and prolonged exercise, exercise- and hypoxia-induced dehydration, or sleep deprivation [1,15,16]. However, the impact of these factors on cognitive performance during mountaineering has rarely been examined. Regarding the influence of exercise, previous investigations have mainly examined the effects of acute exercise on cognitive performance, reporting an improvement of cognitive performance as positive effects of steady-state exercise, as well as fatiguing exercise applicable during, immediately following and after a delay of exercise [17–19]. Other studies have indicated that acute low to moderate exercise may attenuate the risk of impaired cognitive function that occurs under hypoxic conditions [20]. In this context, Komiyama et al. [8] reported that acute exposure to a simulated altitude of 2600 m did not exert sufficient stress to impair working memory or executive function during prolonged exercise of 30 min ergometer cycling. In contrast, strenuous and fatiguing exercise has been suggested to impair mental function under normoxic conditions [21]. Additionally, a combination of acute lack of sleep and strenuous exercise during mountain running has been shown to induce adverse effects on cognitive performance, ranging from lengthened response times to serious symptoms such as visual hallucinations [15].

Overall, there is currently conflicting evidence regarding the effects of hypoxia and strenuous and prolonged exercise on cognitive performance. Because little is known about prolonged exercise under hypoxic conditions affecting attentional function, the current study aimed to examine whether hypobaric hypoxia during a mountain climb at Mt. Kilimanjaro negatively affected attentional performance. Furthermore, we examined potential moderators of hypoxia and prolonged exercise. We hypothesized that both hypoxic conditions and prolonged exercise would impair attentional functioning.

## Materials and methods

### Participants

A sample of 80 healthy young adults (male: n = 51, age 25.5 ± 6.0 yrs; female: n = 29, age 24.8 ± 5.9 yrs) volunteered to participate in the study. Participants were recruited through printed and electronic advertisements on notice boards at the German Sports University Cologne and the Ruhr-University Bochum. Prior to participating in this study, the procedure and the goal of the study as well as the possible risks involved in the experimental procedure were explained to volunteers. If participants were eligible and willing to participate, they were invited to select one part of the study to take part in. Participants were assigned to four groups: A) HYP + EX (hypoxia + exercise), B) HYP, C) EX and D) NOR (normoxia). Previous experience with the Frankfurt Attention Inventory—2 (FAIR-2) test, preceding altitude sojourns above 2000 m in the 4 weeks prior to the investigation, neurological disease, psychiatric illness, learning disabilities, alcohol or drug use or any difficulty that could interfere with behavioral or cognitive testing were used as exclusion criteria. To improve comparability, achievement of a high school certificate as a minimum level of education was used as an inclusion criterion. The study was approved by the ethical committee of the medical department of the Ruhr-University Bochum (GER) and by the ethics committee of the German Sports University Cologne (GER). Participants gave their written informed consent after they were informed about all experimental procedures and risks.

### FAIR-2

We used the FAIR-2 [22–24] to assess attentional performance. This attentional measurement instrument was chosen because of its high retest reliability, expressed as a Cronbach’s alpha between 0.85 and 0.91 [22,25]. Thus, this measurement instrument shows no or only a slightly reduced improvement of performance when habituation to the requirements of the test occurs, which is common in tests of attention and vigilance [26,27].

The FAIR-2 measures a range of factors related to attentional performance, including alertness, focused attention, divided attention and vigilance. The task in this pencil and paper test is to discriminate different test items as quickly and accurately as possible. The test items are similar looking signs, varying in the dimensions of shape (rectangular or circular), the number of points within the sign (two or three) and the arrangement of points within the sign. To increase the rigor of our analysis, we employed two alternative versions of the test (A and B). The participants were given 6 minutes to complete the test, consisting of a total of 640 test items. The number of correctly detected targets as well as correctly rejected non-targets was used for analysis of attentional performance. The task covers three main attentional functions, *performance value* (PV), *continuity value (*CV) and *quality value* (QV). The PV reflects the error-corrected estimate of an attentively processed test item as an indicator of processing speed, the CV reflects the continuity of concentration held over the entire test duration, and the QV reflects the accuracy of attention as the amount of attentively made decisions in relation to the total number of decisions [25,28].

### Experimental design

**Fig 1:**
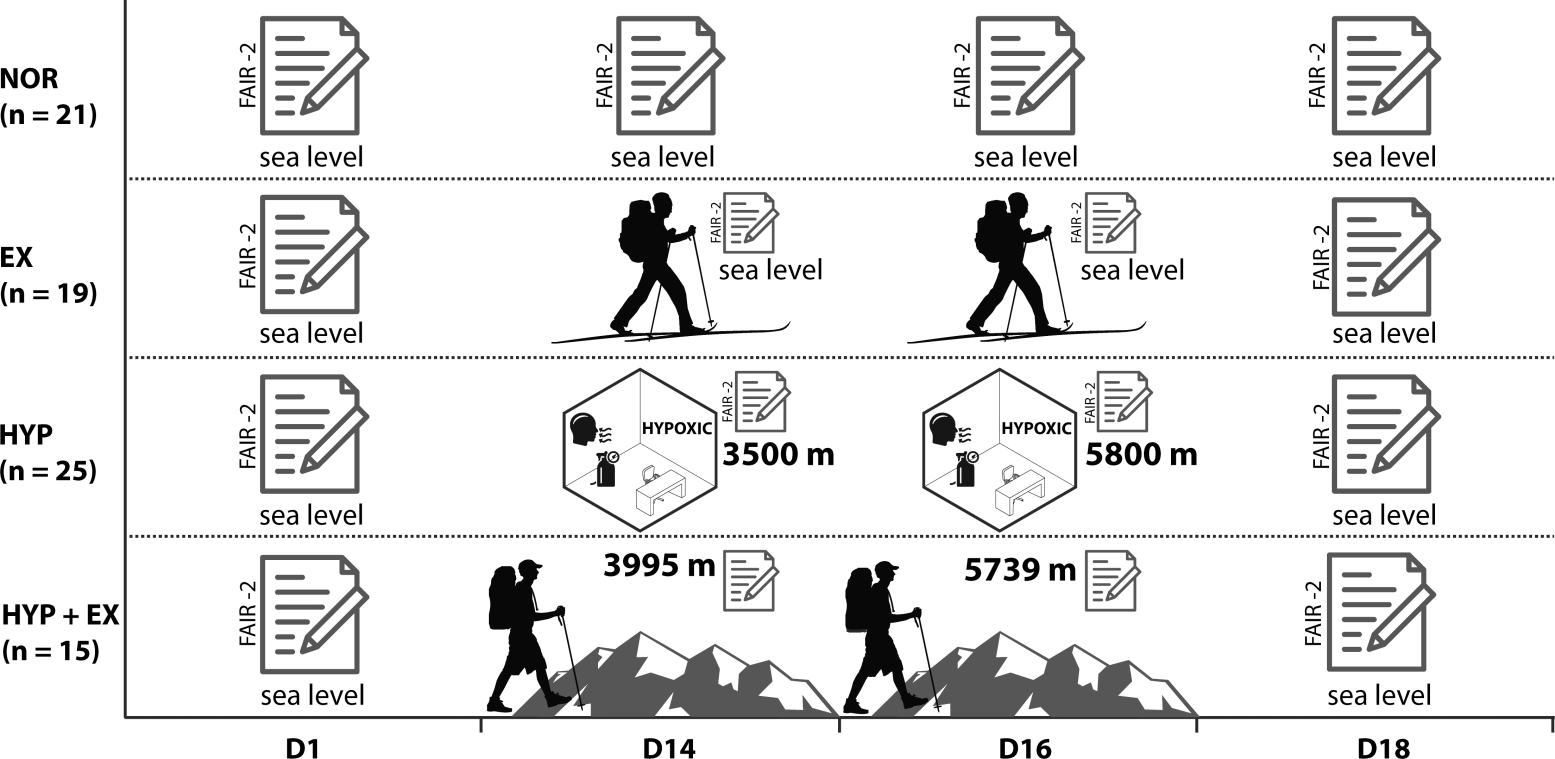
Experimental design.

The study was designed as a quasi-experimental trial. The experimental protocol was completed in 4 testing days over a 16-day period and each group followed the same test protocol. After baseline testing at day 1 (D1), the second test was performed after 2 weeks at day 14 (D14), the third test was performed at day 16 (D16) and the fourth test was performed at day 18 (D18). At D14 and D16 participants were exposed to hypoxia (group B), prolonged exercise (group C) or hypoxia and prolonged exercise (group A). Group D) was a control group, and participants completed all tests under normoxia and at rest. D18 was treated as a follow-up test. Data collection was carried out on the four measurement dates, systematically alternating between version A and B of the FAIR-2, always starting with version A (A-B-A-B) (Fig 1).

#### Group A: HYP+EX

To explore the influence of hypobaric hypoxia and long periods of prolonged exercise on attentional performance during a mountain climb, we recruited 15 participants (male: n = 12, age 27.3 ± 11.6 yrs; female: n = 3, age 32.7 ± 17.6 yrs) while ascending Mt. Kilimanjaro (TZA). In this group, physical health was used as a further inclusion criterion. Therefore, medical history was assessed by self-report using a questionnaire according to the German Association for Sports Medicine and Prevention (DGSP). During the ascent of Mt. Kilimanjaro, no participants took medication for improving acclimatization (e.g., acetazolamide).

The 7-day mountaineering tour involved climbing to 5739 m above sea level (Stella Point, Mt. Kilimanjaro, TZA). Despite its altitude, ascents up Mt. Kilimanjaro are less extreme than typical mountaineering routes found in most other mountain ranges (e.g., the Himalayas, European Alps, Caucasus), and do not involve ice climbing, rock climbing or glacier trekking. In the current study, participants hiked the Lemosho Route in 7 days. The Lemosho Route is the longest route on Mt. Kilimanjaro, offering more acclimatization than other routes. Sleeping heights were set between 2650 m and 4550 m above sea level (Mti Mkubwa Camp 2750 m; Shira1 Camp 3510 m; Barranco Camp 3950 m; Karanga Camp 3995 m; Barafu Camp 4650 m; Millennium Camp 3700 m above sea level). The duration of daily hiking ranged from 3 h on the first day to 14 h during the summit climb.

The self-reported Lake Louise Score (LLS) was used to measure the perceived symptoms of acute mountain sickness (AMS) [29–31]. The questionnaire consists of five questions concerning the characteristic symptoms for AMS: headache, gastro-intestinal discomfort (vomiting, nausea, loss of appetite), fatigue and/or weaknesses, dizziness, and insomnia. Participants answered the questions using a 4-point Likert scale (0–3) and the presence of headache in combination with the sum score was used to detect AMS. Participants filled out the LLS scoring sheet every morning and every evening during the 7-day mountain climb and immediately before assessment of the FAIR-2. The FAIR-2 related LLS scores were used for further analyses. In addition, we measured peripheral oxygen saturation using a pulse-oximeter (PO-100, Pulox Cologne, GER) at the same time points.

Baseline attentional performance was measured at rest under normoxic conditions (D1; 154 m above sea level) while sitting at a desk in a quiet room. The second test was conducted after the fourth day of mountaineering (D14; 3995 m above sea level). The following testing was conducted on the sixth day of mountaineering at Stella Point (D16; 5739 m above sea level). For both tests, participants were seated in a quiet area on the ground, and completed the FAIR-2 using a clipboard as a base for writing. The follow-up testing (D18) was completed again at rest and under normoxic conditions (812 m above sea level) while sitting at a table in a quiet outdoor area.

#### Group B: HYP

Group B consisted of 25 participants (male: n = 19, age 24.7 ± 3.1 yrs; female: n = 6, age 22.7 ± 2.3 yrs). In this group, physical health was also used as an additional inclusion criterion. Therefore, we used the same self-report questionnaire according to the DGSP as already described for group A. To assess the influence of simulated altitude on attentional performance, the FAIR-2 test was conducted at three different heights (D1: 53 m; D14: 3800 m; D16: 5800 m; D18: 53 m). In testing on D1 and D18, participants completed the test instructions and the FAIR-2 test within 15 minutes under normoxic conditions while sitting at a desk in a quiet room. At D14 and D16, participants were exposed to simulated, normobaric hypoxia in a hypoxic chamber.

Participants completed a 15-minute resting wash-in period while inspiring oxygen-reduced air. Peripheral oxygen saturation was measured using a pulse-oximeter (PO-100, Pulox Cologne, GER). The FAIR-2 test was completed within 6 minutes following the 15-minute wash-in period. The LLS was used to measure symptoms of acute hypoxia [2,29,32]. Participants filled out the LLS scoring sheet right after finishing the FAIR-2 test.

The D14 test was performed in a hypoxic chamber equipped for altitude simulations up to 3500 m. Participants entered the hypoxic chamber, in which the oxygen concentration was reduced to 13.5%, equivalent to an altitude level of 3500 m, using hypoxic generators (Regalia 10, SeQual Technologies, CA, USA; Everest Summit II, Hypoxico, NY, USA). A carbon dioxide absorber (Purabox CS 2210, SK Engineering, Kiel, GER) was used to maintain the CO_2_ level constantly below 0.5%. Throughout the test on D16, participants entered the same chamber to ensure a similar test setting but wore a silicon mask connected to an oxygen-depleting respiratory system (Everest Summit II, Hypoxico, NY, USA) and breathed through a low resistance two-way respiratory valve. The inspired air (FiO_2_) consisted of 10.0% O_2_, equivalent to an altitude of 5800 m.

The room temperature in the hypoxic chamber was kept at a constant level of 22–24°C using an air conditioning system (PAC FX 550 ECO, DeLonghi, Treviso, IT) for both tests. O_2_ and CO_2_ levels were observed continuously using a multi-gas detector (X-am 7000, Dräger, Lübeck, GER).

#### Group C: EX

To examine the influence of long periods of prolonged exercise on attentional performance, 19 participants (male: n = 9, age 24.4 ± 1.3 yrs; female: n = 10, age 24.0 ± 2.3 yrs) completed a 7-day backcountry ski hiking tour in the high plateaus Setesdal- and Sirdalheiane in the south of Norway. Sleeping altitude and the altitude of the ski hiking routes largely ranged between 700 and 1000 m above sea level. The highest point reached during the tour was 1200 m above sea level. Because no hypoxia-induced physiological changes were expected at such low altitudes, this group was assumed to react as if they were under normoxic conditions. The distances for each day were set between 14 and 29 kilometers, with up to 500 m of positive altitude change per day, resulting in a daily duration of ski hiking that ranged between 4 h and 10 h, including rests. Participants slept in self-catered huts, and carried backpacks weighing between 15 and 20 kg. Attentional performance was measured for baseline testing at rest (D1) while participants sat at a desk in a quiet room (53 m above sea level). The following tests were conducted directly after ski hiking on the fourth (D14; 940 m above sea level) and sixth day (D16; 750 m above sea level). Tests were conducted after 20 km and 7.4 hours (D14) and 17 km and 6.2 hours of ski hiking including rests. The FAIR-2 was carried out while participants sat at tables in the common room of the hut. If there were other visitors in the common room, they were asked to leave the room for 15 minutes, or to remain quiet during testing, to ensure a quiet testing environment. Follow-up testing (D18) was completed at rest again (50 m above sea level) while participants sat in a quiet room.

#### Group D: NOR

A control group consisting of 21 participants (male: n = 11, age 24.7 ± 3.1; female: n = 10, age 24.4 ± 2.2) was recruited. Participants performed the FAIR-2 test under normoxic conditions (53 m above sea level) in the afternoon following the same test protocol (D1, D14, D16, D18) as in groups A–C. Participants sat at a desk in a quiet room and completed the test instructions and the FAIR-2 test within 15 minutes under normoxic conditions (53 m above sea level).

### Statistical analysis

Data are presented as means ± SDs. The Shapiro–Wilk test was used to identify all departures from the normal distribution. *Internal validity*: To detect potential differences in pre-test performance values (PV, CV) between groups (HYP + EX, HYP, EX, NOR), one-way ANOVAs were performed, followed by a post-hoc comparison of the means using Bonferroni correction. When the variables were not normally distributed (QV), the Kruskal–Wallis test was used to identify differences between groups. Dunn-Bonferroni tests were used for post-hoc comparisons of the means with corrections for multiple tests to retain an alpha level of 0.05. Cohen’s d (*d*, *f*) was used to calculate effect sizes [33]. *Learning effect*: Repeated measures ANOVAs were used to test whether the repeated measurement of the FAIR-2 exerted an influence on the attentional functions of PV and CV and to determine whether test results revealed a learning effect over time in the control group (NOR). Greenhouse-Geisser adjustments were used to correct for violation of the assumption of sphericity. Two tailed t-tests were used for post-hoc comparisons of the means with Bonferroni corrections for multiple t-tests to retain an alpha level of 0.05. Cohen’s d (*f*) was used to calculate effect sizes [33]. When variables were not normally distributed (QV), the Friedman test was used to identify differences between tests (D1, D14, D16, D18). Dunn-Bonferroni tests were used for post-hoc comparisons of the means with corrections for multiple tests to retain an alpha level of 0.05. Kendall’s *W* (coefficient of concordance) was used to interpret the effect sizes. Pearson’s product-moment correlations were calculated for retest reliability of the attention functions PV, CV, and QV (NOR) between D1 and D14. *FAIR-2 performance development*: We calculated performance changes in PV, CV, and QV (Δ1 = D14 –D1; Δ2 = D16 –D14; Δ3 = D18 –D16) for further analysis. Because variables were not normally distributed (ΔPV, ΔCV, ΔQV), Kruskal–Wallis tests were performed to compare the performance change between groups (HYP + EX, HYP, EX, NOR) for Δ1, Δ2 and Δ3. Dunn–Bonferroni tests were used for post-hoc comparisons of the means with corrections for multiple tests to retain an alpha level of 0.05. Cohen’s d (*d*) and correlation coefficients (r) were used to calculate effect sizes for the overall effect and the post-hoc effects, respectively [33]. *Blood oxygen saturation and Lake Louise Score*: Paired sample *t-*tests were calculated for pairwise comparisons of s_p_O_2_ for group A (HYP + EX) and B (HYP) at 3800 m and 5800 m. Students *t*-tests were calculated for differences in s_p_O_2_ between group A (HYP + EX) and B (HYP). Wilcoxon tests were used to test differences of LLS between 3800 m and 5800 m for group A (HYP + EX) and B (HYP). Mann-Whitney tests were used to test differences in LLS between group A (HYP + EX) and B (HYP). Cohen’s d (*d*) and correlation coefficients (r) were used to calculate effect sizes. The relationships between attentional functions and s_p_O_2_ were analyzed using Pearson’s product-moment correlations. The relationships between attentional functions and LLS were analyzed using Spearman rank-order correlations. The alpha level was set at p ≤ 0.05, and all analyses were conducted using SPSS 25 (IBM, Armonk, NY, USA.).

## Results

### Internal validity

There were no significant differences between groups in the performance parameters PV (F = 0.046, p = 0.987), CV (F = 0.083, p = 0.969) and QV (χ2 = 2.564, p = 0.987).

### Learning effect

#### Group D: NOR

**Fig 2:**
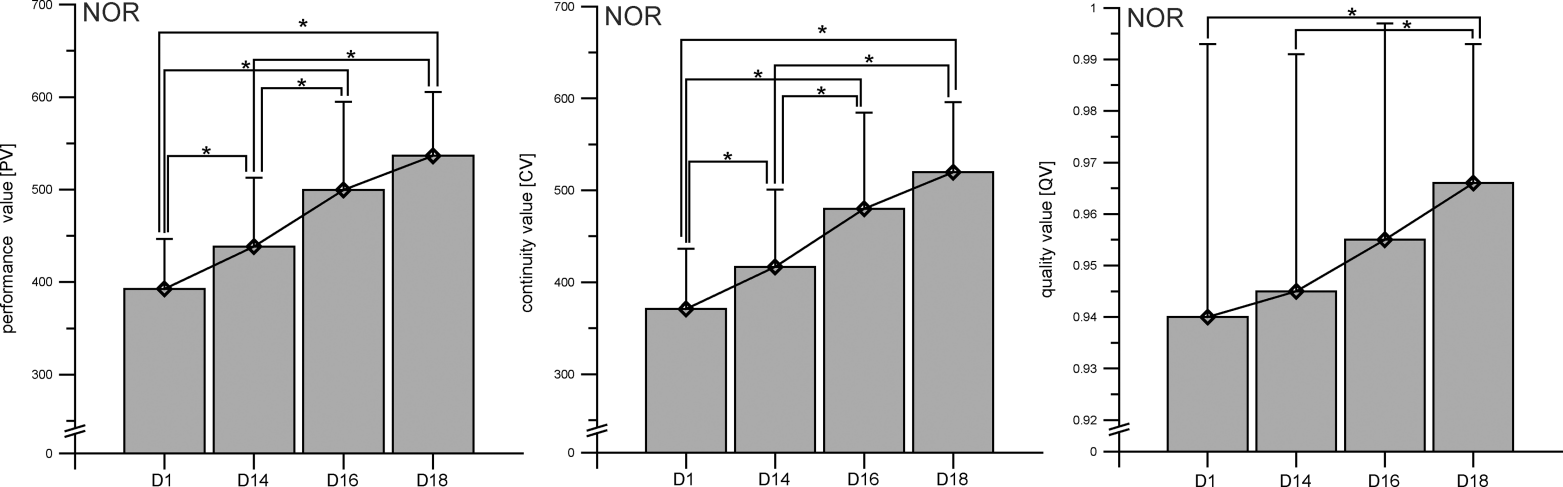
Learning effect. Increase in attentional performance parameters performance value (PV), continuity value (CV), and quality value (QV) for four testing points (day1, day14, day16, day18) in the control group (NOR; n = 21). Data points represent means ± SD. See METHODS for further details. *p ≤ 0.05.

Comparison of the values of PV, CV and QV over the four testing points in group D [NOR] revealed a clear influence of the repetition of measurement on attentional performance for all factors (PV: F = 62.9, p = 0.000, *f* = 1.775; CV: F = 59.9, p = 0.000, *f* = 0.624; QV: **χ**^**2**^ = 16.721, p = 0.001, *W* = 0.265) (Fig 2). Because these results indicated a learning effect, we calculated the performance change (Δ) for further analyses (Δ1 = D14 –D1; Δ2 = D16 –D14; Δ3 = D18 –D16) for all groups. Additionally, comparison of the attention functions PV, CV, and QV on D1 and D14 revealed significant correlations for PV (r = 0.720, p = 0.000), CV (r = 0.769, p = 0.000), and QV (r = 0.906, p = 0.000).

### FAIR-2 performance development

We found very strong evidence of a difference between the mean ranks of at least one pair of groups for PV (Δ2: χ^2^ = 25.689, p < 0.001, *d* = 1.305; Δ3: χ^2^ = 21.858, p < 0.001, *d* = 1.149), CV (Δ2: χ^2^ = 24.965, p < 0.001, *d* = 1.275; Δ3: χ^2^ = 18.958, p < 0.001, *d* = 1.031), and QV (Δ2: χ^2^ = 9.999, p = 0.019, *d* = 0.637).

#### Group A: HYP + EX

**Fig 3:**
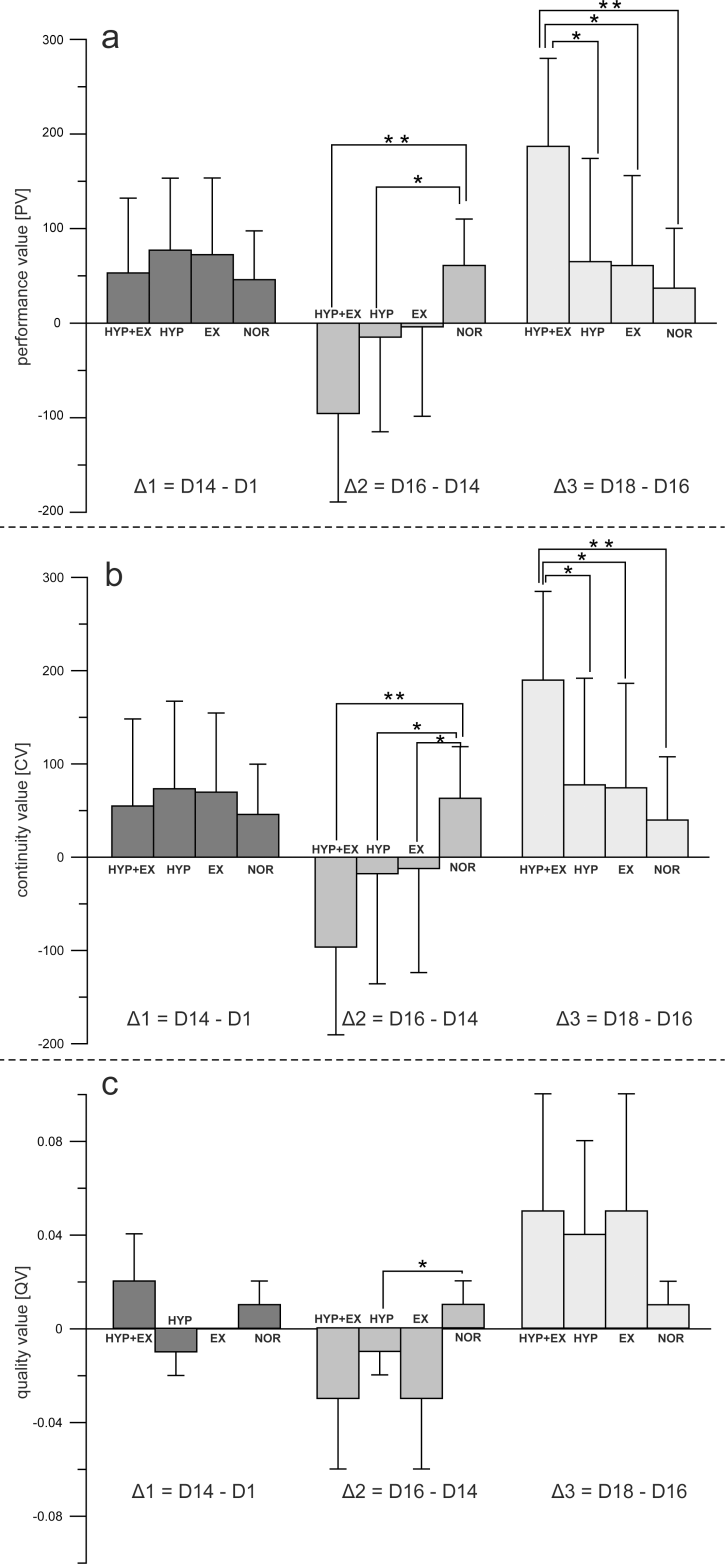
**FAIR-2 performance developments** (Δ1, Δ2, Δ3) of attentional performance parameters a) performance value (PV), b) continuity value (CV), and c) quality value (QV) for the four experimental groups HYP+EX, HYP, EX, and NOR. Bar charts are means ± SD. See METHODS and RESULTS for further details. *p ≤ 0.05, **p ≤ 0.001.

Post-hoc comparisons showed that participants in the HYP + EX group exhibited significantly higher performance changes for selective attention (PV) compared with the NOR group in Δ2 (p = 0.000, *r* = 0.827) (Fig 3A). Returning to sea level resulted in a significantly greater performance change of PV in Δ3 compared with the NOR (p = 0.000, *r* = 0.753), HYP (p = 0.005, *r* = 0.529) and EX (p = 0.003, *r* = 0.588) groups (Fig 3A). In addition, we found a decrease for continuity of attention (CV) compared with the NOR group in Δ2 (p = 0.000, *r* = 0.807) and an increase of CV compared with all groups in Δ3 [NOR (p = 0.000, *r* = 0.711), HYP (p = 0.022, *r* = 0.459), EX (p = 0.009, *r* = 0.527)] (Fig 3B). We found no effects on self-control (QV) (Fig 3C).

#### Group B: HYP

The HYP group exhibited a significantly higher performance change in Δ2 compared with the NOR group for PV (p = 0.006, *r* = 0.482), CV (p = 0.004, *r* = 0.499), and QV (p = 0.031, *r* = 0.413) (Fig 3).

#### Group C: EX

The EX group showed a significantly decrease in Δ2 compared with the NOR group for CV (p = 0.042, *r* = 0.416). We found no effect for the factors PV and QV (Fig 3).

### Blood oxygen saturation

#### Group A: HYP + EX and Group B: HYP

**Fig 4:**
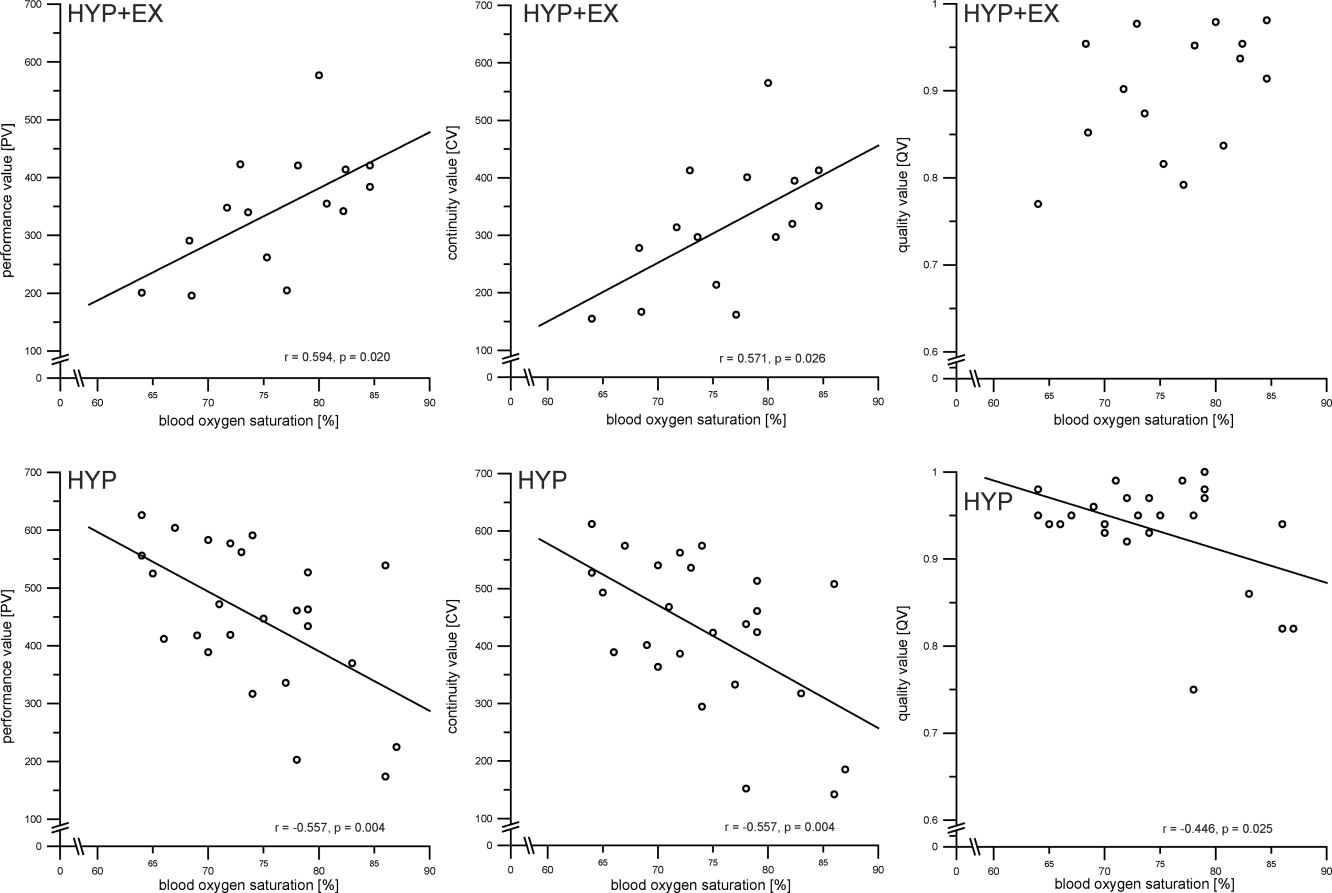
Pearsons’ product-moment correlations for relations of attentional performance parameters performance value (PV), continuity value (CV), and quality value (QV) and blood oxygen saturation (s_p_O_2_) for both hypoxia groups (HYP+EX and HYP) at d16 (5800 m). Data points represent individual values. *p ≤ 0.05.

Under normobaric hypoxic conditions (HYP), oxygen saturation (s_p_O_2_) was lower at 5800 m (74.32 ± 6.84%) compared with values at 3800 m (87.44 ± 4.03%) (p = 0.000, *d* = -2.337). We found similar results under hypobaric hypoxic conditions (HYP + EX) with significant lower s_p_O_2_ values at 5800 m (76.27 ± 6.35%) compared with 3800 m (83.13 ± 4.44%) (p = 0.000, *d* = -1.252). Differences between HYP + EX and HYP were found for 3800 m (HYP + EX: 83.13 ± 4.44; EX: 87.44 ± 4.03%; p = 0.005, *d* = 1.030), but not for 5800 m (HYP + EX: 76.27 ± 6.35; EX: 74.32 ± 6.84%; p = 0.369). In addition, s_p_O_2_ values were inversely correlated with the attention functions PV, CV, and QV at 5800 m for HYP (r = −0.557, p = 0.004 for PV; r = −0.557, p = 0.004 for CV; r = −0.446, p = 0.025 for QV), but positively correlated with the functions PV and CV for HYP + EX (r = 0.594, p = 0.020 for PV; r = 0.571, p = 0.026 for CV) (Fig 4). The analysis revealed no relationship between s_p_O_2_ and the attentional functions PV, CV, and QV at an altitude of 3800 m.

### Lake louise score

#### Group A: HYP + EX and Group B: HYP

Participants in the HYP + EX group showed significantly higher LLS values at 5800 m (5.1 ± 3.2) compared with 3800 m (1.9 ± 2.4) (p = 0.009, *r* = 0.674). For HYP participants, LLS values were significantly higher at 5800 m (2.2 ± 2.2) compared with 3800 m (0.8 ± 0.9) (p = 0.007, *r* = 0.540). In addition, we found significant higher LLS values at 5800 m for the HYP + EX group (5.1 ± 3.2) compared with the HYP group (2.2 ± 2.2; p = 0.004, *d* = 1.003). No differences were found between the HYP + EX group (1.9 ± 2.4) and the HYP group (0.8 ± 0.9) at an altitude of 3800 m. In addition, LLS values were strongly correlated with the attentional functions PV and CV at 5800 m for HYP + EX participants (r = 0.602, p = 0.018 for PV; r = 0.627, p = 0.012 for CV), or for QV at 3800 m (r = 0.527, p = 0.044 for QV). For HYP participants, we found a moderate negative correlation between the attentional function QV and LLS values (r = −0.398, p = 0.049 for QV). The analysis revealed no relationship between LLS and the attentional functions PV, CV and QV for the HYP group at 3800 m.

## Discussion

We hypothesized that attentional performance would be impaired during a mountain climb up to 5800 m because of the effects of hypoxic conditions and prolonged exercise. In addition, we predicted that the potential moderators *hypoxia* and *prolonged exercise* would influence attention.

The main findings of this study were as follows:

1. Exposure to hypoxic conditions negatively affected attention at a height of 5800 m, but not at a height of 3800 m. Specifically, the attentional functions PV and CV were significantly impaired, compared with participants tested under normoxic conditions. This result supports previous studies reporting impairment of cognitive functions at high altitudes [1,20,34–37], but not at moderate altitudes [8,38]. However, some previous studies did not show decreased cognitive performance even at high altitudes [39–41], indicating a possible relationship between the state of acclimatization and the severity of cognitive impairment under hypoxic conditions. Referring to the classification of moderate altitudes between 2000 and 3000 m, high altitudes between 3000 and 5500 m and extreme altitudes above 5500 m, we considered 3800 m as a high altitude and 5800 m as an extreme altitude [42]. One possible interpretation of the current findings is that impairment of attentional functioning only occurs at extreme altitudes, and not at high altitudes. Regarding the acclimatization protocol in the present study for group A (HYP + EX), we expected that acclimatization would play an important role in the severity of cognitive impairment. Before testing at 3950 m, participants undertook 3 days of mountain climbing between 2750 and 3950 m, whereas for the testing at 5739 m upon arrival at Stella Point on Mt. Kilimanjaro, participants had completed nearly 1800 m of climbing within only 24 h. Thus, it would be reasonable to assume that participants were in a more acclimatized state at the second test (3800 m) compared with the third test (5800 m). Thus, we conclude that attentional performance at 3800 m may not have been impaired in participants in the HYP + EX group because they were better acclimatized than at 5739 m. Another finding of this study was that the number of correct test items (PV) and the continuity of attentional performance was decreased in extreme hypoxic compared with normoxic conditions, whereas task accuracy was unaffected by hypoxic conditions. This result is consistent with previous studies [8,43,44], which additionally found an association between cerebral oxygenation and task performance in cognitive testing under hypoxia and suggest that reduced task speed and performance might reflect a compensatory effect to maintain accuracy of attention [44,45].
2. The moderator *prolonged exercise* had no significant impact on attentional performance. However, we found significant reduced attention performance during the mountain climb up to 5800 m compared with all other groups, suggesting that additional moderators besides hypoxic conditions may influence attentional performance during high-altitude mountain climbing. There are several possible explanations for the diminished cognitive performance despite reduced O_2_ intake under hypoxic conditions. First, participants in an earlier study exhibited serious cognitive impairments in reaction time and errors of commission during an extreme endurance event, which was attributed to a combination of sleep deprivation and strenuous exercise [15]. Moreover, it has been reported that the worsening of sleep due to hypoxia impairs cognition after 24 h of hypoxia in a normobaric chamber simulating an altitude of 4500 m [1], and restricting sleep to between 3 and 6 h has been shown to increase attentional dysfunction [16]. Thus, hypoxia and sleep deprivation may both contribute to attentional deficits in mountaineers at high altitude. It has also been proposed that the relatively low temperatures at high altitude affect brain functions [46]. Attentional encoding processes are impaired when exposed to thermal stress (e.g., cold) [16]. Another possible explanation is that cognitive impairment is not only caused by a decreased O_2_ intake, but also by the reduced barometric pressure at high altitude. Reduced average barometric pressure caused by weather changes has been shown to be associated with headache and the frequency of migraine [47,48], suggesting that changes in barometric pressure influence brain function. One major limitation of the current study is the lack of data regarding secondary environmental influences in addition to strenuous and fatiguing exercise. More research is required to determine whether an interaction exists between reduced barometric pressure at altitude, low temperatures, dehydration, or sleep deprivation and attentional impairment. To isolate the effects of hypoxia and exercise, an appropriate experimental design might involve an additional control group under short-term hypobaric hypoxic conditions (e.g., ascending to the same altitude by car, gondola, or plane).
3. The results revealed a negative correlation between oxygen saturation and all attentional functions (PV, CV and QV), as well as a negative correlation between the physiological symptoms associated with hypoxia (LLS) and QV in the short-term normobaric hypoxia group (HYP) at an altitude of 5800 m. In addition, we found lower blood oxygen saturation and higher LLS values at 5800 m than 3800 m, as expected, in the HYP group. The negative correlation between LLS and QV suggests that the accuracy of attention (QV) decreases with perceived physiological symptoms caused by acute hypoxia. However, although the overall effect showed impaired attentional functions for Δ2, subjects with lower oxygen saturation exhibited better attention performance at 5800 m. This finding was somewhat unexpected and contradictory to previous investigations, because the severity of cognitive impairment mainly depends on the severity of hypoxia [7,49,50]. Moreover, recent studies suggest that the severity of s_p_O_2_ and P_a_O_2_ may be a key predictor of cognitive impairments under hypoxic conditions [49,50]. This is supported by Komiyama et al. [51], who investigated executive cognitive functions during exercise under severe hypoxia. The authors hypothesized that there would be an improvement in cognitive functions during exercise under severe hypoxia, but finally reported an attenuation of this improvement in individuals exhibiting a greater decrease in s_p_O_2_. One possible explanation for this unexpected finding in the present study and its inconsistency with previous study findings is that visual contrast sensitivity is particularly enhanced during acute, short-term hypoxia, as reported in earlier investigations [45,46]. The FAIR-2 test is based on visual discrimination of different test items. Thus, an improvement in FAIR-2 performance may have been caused by enhanced visual contrast sensitivity only occurring under severe hypoxic conditions.

In contrast, the long-term hypobaric hypoxia group (HYP + EX) exhibited a positive correlation between blood oxygen saturation and attentional functions at 5800 m, such that higher oxygen saturation was related to greater selective attention (PV) and continuity of attention (CV). This finding may reflect the previously reported relationship between the state of acclimatization and the severity of cognitive impairment under hypoxic conditions [52]. It could be assumed that higher oxygen saturation reflects better acclimatization to altitude, and therefore results in improved cognitive abilities compared with less acclimatized individuals. Preservation of working memory abilities was reported in a recent study of properly acclimatized mountain climbers [53] and the effects of acclimatization may help to consolidate cognition during altitude exposure [50,53]. Moreover, the potential positive influence of exercise on cognitive function during mountain climbing compared with hypoxic exposure at rest should also be considered. However, this hypothesis requires further investigation to clarify the possible underlying mechanisms.

Unlike the normobaric hypoxic group (HYP), the HYP + EX group exhibited positive correlations between LLS and the attentional functions PV and CV. Thus, the rate of work (PV) and continuity of attention (CV) increased with perceived physiological symptoms associated with hypoxia. One potential explanation for the differences between the HYP group and the HYP + EX group is that there is a different impact of normobaric versus hypobaric hypoxic exposure on cognitive function. However, it has already been shown that the type of hypoxic condition (hypobaric vs. normobaric hypoxic) does not represent a significant moderator for cognitive impairment caused by hypoxic conditions [50]. In addition, it has been suggested that the use of the self-reported LLS leads to different assessments of AMS in hypobaric hypoxia compared with normobaric hypoxia [54]. Thus, one limitation of the present study involves the shortcomings of the LLS. For example, there may be difficulties concerning interpersonal psychological aspects, the specificity of symptoms for AMS, and the understanding of the terminology used in the self-reported questionnaire. Furthermore, the symptoms of sleep and headache should also be considered. Although sleep quality is not strongly related to other AMS symptoms, and its removal from the LLS has therefore been recommended [55,56], headache is generally considered a leading symptom of AMS, although West [57] proposes that headache should not be a required symptom for the diagnosis of AMS. However, the LLS is still a recommended AMS self-report questionnaire because of its simplicity and rapidity [32], and is the most popular questionnaire in current use [58]. Several recent studies have strongly recommended alternative subjective assessments of acute mountain sickness, including the Visual Analog Scores (VAS) [58,59] or the Environmental Symptoms Questionnaire (ESQ) [60]. These measures should be considered in future field studies investigating perceived AMS symptoms during high altitude mountain climbs. Another explanation for the differences between the HYP group and the HYP + EX group, and a concurrent shortcoming of the present study, is the difference in hypoxic exposure duration. While the HYP + EX group was exposed to prolonged hypoxia, the HYP group was only exposed to acute hypoxia. As already mentioned, acclimatization may have affected our results for attentional performance measurements in the HYP + EX group; therefore, direct comparisons between HYP and HYP + EX conditions must be made with caution. The different duration of hypoxic exposure in combination with other environmental conditions (e.g., temperature, humidity) may also explain the group differences in responses. Additionally, the different test conditions for HYP at D14 (hypoxic chamber) and D16 (silicon face mask connected to an oxygen-depleting respiratory system) may have been a potential limitation of the present study. However, participants reported no constraints in completing the FAIR-2 when wearing the silicon mask. We therefore conclude that the different test settings did not influence cognitive testing results in this study.

In addition, we found limited or no attentional impairment at the lower altitude of 3800 m for both hypoxic groups (Fig 3). This finding potentially supports the results of Pilmanis et al. [61], who reported a minimal influence of low-grade hypoxia on cognitive performance. The authors found small degradations in cognitive performance at simulated altitudes of 2438 m and 3658 m, but for only two of the cognitive tasks tested. In addition, Griva et al. [13] initially found a levelling or even an improvement in cognitive abilities at moderate altitudes (1300 m and 3500 m) compared with baseline measurements, which declined with ascents to higher altitudes (5300 m). These findings are in accord with the established notion that the severity of impairment depends on the severity of hypoxia and the rate of ascent [7].

To the best of our knowledge, this is the first study to describe a relationship between oxygen saturation as well as physiological symptoms (LLS) and a hypoxia-induced decline in attentional functioning. A previous study reported that PaO_2_ was a key predictor of cognitive performance [62]. However, Pramsohler et al. [63] also observed impaired reaction times after a single night sleeping at a simulated altitude of 3500 m, but no relationship between cognitive impairment and low oxygen saturation. They concluded that the causality between low oxygen saturation and cognitive functioning remains unclear. Therefore, we suggest that further work is required to elucidate the interrelations of cognitive functions under conditions of chronic hypoxia and acute hypoxia and blood oxygen saturation values, as well as LLS values. The current results further suggest a correlation between LLS or blood oxygen saturation and attentional function at extreme altitudes.

4 Finally, the current findings revealed a learning effect of completing the FAIR-2 test four times over an 18-day period. We chose the FAIR-2 test because of its high retest reliability, as described in the test instructions, with a Cronbach’s alpha of 0.81 for PV and CV and 0.73 for QV after 2 weeks [22], and Cronbach’s alpha values between 0.85 and 0.91 reported in recent studies [24,25]. We thereby expected only a small improvement of performance resulting from a habituation to the requirements of the test. However, habituation to test requirements is common in tests of attention and vigilance [26,27]. In the current study, we found indeed a significant increase across repeated measurements of the FAIR-2. Further, the retest reliability for group D was only 0.720 for PV and 0.769 for CV, whereas it was 0.906 for QV. One limitation that should be considered is the relatively small sample size in the current study (n = 21) compared with the recommendations in the test instructions (n = 53) [22]. Thus, we conclude that more research is needed to verify the reported high retest reliability of the FAIR-2 test. We further suggest that habituation sessions may help to avoid learning effects during the main experiment. Moreover, the limitations of the FAIR-2 and the assessment used in the current study should also be considered. Because our ability to carry equipment to high altitude was restricted, and our power supply had limited reliability, we decided to assess attentional functions using written pencil and paper tests, which have been shown to be associated with greater variability than computerized tests [39]. Another potential limitation of the study is the quasi-experimental design and the selection bias that might have occurred because participants were free to choose one of the four experimental group assignments. However, there was no inequality between experimental groups for the pre-test values of PV, CV and QV. We therefore concluded that the group comparisons did not threaten the internal validity and that differences in the performance tests could be attributed to the variables hypoxia (HYP) and exercise (EX).

## Conclusion

The present findings suggest that attention is impaired during extreme normobaric and hypobaric hypoxic exposure, with the strongest effects occurring during mountain climbs at extreme altitudes. Moreover, we conclude that greater cognitive impairment under hypobaric hypoxia during a mountain climb compared with normobaric hypoxia at rest does not appear to be caused by prolonged exercise. There are significant methodical testing challenges when testing for attentional impairments between simulated and real world altitude conditions. Thus, additional studies are required to standardize these methodical factors and identify additional factors besides hypoxia that negatively impact attention during mountain climbs, such as sleep deprivation, dehydration, or low temperatures at high to extreme altitudes.
